# How would you describe a mentally healthy college student based on Chinese culture? A qualitative research from the perspective of college students

**DOI:** 10.1186/s40359-024-01689-7

**Published:** 2024-04-15

**Authors:** Mingjia Guo, Xiaoming Jia, Wenqian Wang

**Affiliations:** School of Humanities and Social Sciences, Beijing Institute of Technology, Beijing, China

**Keywords:** College students, Mental health, Qualitative research, Mental health promotion, Chinese culture

## Abstract

**Background:**

Promoting college students’ mental health remains a significant concern, necessitating a clear understanding of what constitutes good mental health. Variations in the conceptualizations of mental health across cultures, typically derived from academic and authoritative perspectives, have overlooked insights from laypeople. This study aims to investigate the characteristics of mentally healthy college students within Chinese cultural contexts, emphasizing perspectives provided by college students themselves.

**Methods:**

Undergraduates with self-reported mental health scores ≥ 7 were randomly selected for in-depth interviews. The sample (*N* = 17, 59% female) had a mean age of 20.82 ± 1.33 years and represented diverse regions, backgrounds, and academic fields. Thematic analysis was used in the analysis of the qualitative data, involving initial coding to identify 168 manifestations of mental health among college students, followed by categorizing them into 18 characteristics through focused coding. These characteristics were then organized into five themes via core coding. The Delphi method was utilized to validate the themes with 3 experts, ensuring the trustworthiness of the final findings.

**Results:**

Eighteen characteristics of mentally healthy college students emerged from the interviews, categorized into 5 themes: (1)Value Pursuit (i.e. Having a sense of responsibility and mission and being willing to dedicate oneself to the country at any time.); (2)Life Attitude (i.e. Staying positive and having the ability and quality to cope with hardships.); (3)Interpersonal Ideals (i.e., Showing filial respect to parents appropriately.); (4)Behavior Ability(i.e., Studying diligently and learning well.); and (5)Self-cultivation (i.e., Possessing good qualities advocated by Confucianism, Buddhism, and Taoism coexist harmoniously.). Most of these characteristics directly reflect traditional Chinese culture or culture that has changed with the times. At the same time, some are a reflection of modern Chinese new culture.

**Conclusions:**

On the whole, the characteristics of mentally healthy college students are diverse and with rich connotations, focusing on the individual’s relationship with the country, family, and others, and are good expressions of Chinese cultural features, such as the balance of Yin and Yang, the coexistence of Confucianism, Buddhism, and Taoism, and highlight moral attributes. In essence, these traits hold profound importance in advancing the mental health of Chinese college students.

**Supplementary Information:**

The online version contains supplementary material available at 10.1186/s40359-024-01689-7.

## Background

The period of undergraduate study is vital for individual development, physical and mental growth, knowledge reserve, and health literacy development. For undergraduate students, they are in the process of transitioning from late adolescence to early adulthood, navigating various physical, psychological, and social changes [1]. After entering the university, undergraduates, especially first-year students, are prone to various maladaptation problems due to changes in their living and learning environments [2]. Notably, a recent nationwide survey of 48,789 undergraduate students from 31 provinces and cities of China showed that 24.17% of undergraduates were at risk of depression, and 49.58% were at risk of anxiety [3].

Some studies have shown that these psychological problems are related to culture. As a Chinese proverb goes, “Nothing is more important than learning.” Before university, Chinese students focused solely on their studies, with their parents managing all aspects of life [4]. Consequently, they may lack the ability to independently resolve problems, particularly when confronted with many challenges in university life, often feeling helpless. Furthermore, admission to university is considered an honor to ancestors and a source of pride for parents in Chinese culture [5]. Attaining good grades and securing an ideal career post-graduation are seen as ways for college students to fulfill their filial duties, like supporting their parents, thus imposing familial and communal pressures.

Cultural influences also play a role in the mental health of college students. Wang et al. (2016) investigated how traditional Chinese philosophies—such as relationship harmony (advocated by Confucianism), dialectical coping (from Taoism), and non-attachment (rooted in Buddhism)—impact college students’ mental health. Their research demonstrated these philosophies’ negative correlation with psychological distress and negative emotions while displaying positive correlations with self-esteem, positive emotions, meaning of life, and happiness [6]. Another study indicated that Chinese college students scoring higher in Zhongyong thinking exhibit lower anxiety and depressive symptoms, along with higher self-esteem and life satisfaction, versus those with lower scores [7].

Since culture and mental health are mutually embedded [8], different cultures may interpret the same things differently. For instance, in Western cultures, pursuing a college education is often viewed as an individual pursuit, whereas in collectivist China, but in China, higher education is commonly sought to elevate social status and offer enhanced financial support to parents, such as securing a comfortable retirement home. In times of conflict, individuals in Chinese society tend to adopt the principle of “taking a step back and yielding vastness and spaciousness to others” [9], prioritizing long-term harmony over immediate gains by favoring conflict avoidance over confrontation. The values of “harmony is precious” and the practice of “forbearance” are revered in China, whereas in Western societies, it may be considered unhealthy, with individuals opting for direct expression of discontent [10].

In China, only 8% of the population hold bachelor’s degrees [11], and college students are seen as the nation’s hope and future [12], underscoring a heightened focus on their mental health. To enhance the mental health of Chinese college students effectively, it is imperative to grasp the cultural nuances defining mental health across various contexts.

Mental health has always been a focus in the field of psychology. Researchers from diverse backgrounds have extensively investigated mental health within various cultural frameworks. This includes the development of nuanced interpretations and pertinent theories regarding mental health across different cultural settings [13, 14]. Moreover, scholars have localized measurement tools through meticulous adaptations [1, 15–17] and delved into understanding the impact mechanisms between mental health and its associated determinants [18, 19]. In terms of the connotation of mental health, aside from the various approaches of counseling and psychotherapy have their interpretations and definitions of mental health, various organizations and scholars have also put forward different perspectives of mental health from multifaceted viewpoints, clearly demonstrating the impact of culture.

According to the Concise Encyclopaedia Britannica, *mental health* is defined as “the state of optimal functioning of the individual psyche within the limits of its own and environmental conditions, but not as an absolute state of perfection” [20]. Meeks and Heit describe *mental health* as “the ability to perceive and express one’s emotions and state of mind; mental health is the ability to accept reality as it is” [21]. Meanwhile, Ryan and Deci propose that *mental health* involves “the ability to feel effective and agile, e.g., to have full self-fulfillment” [22]. The World Health Organization defines *mental health* as “a state of well-being in which the individual realizes his or her abilities, can cope with the normal stresses of life, can work productively and fruitfully, and can make a contribution to his or her community” [23]. These definitions illustrate how Western culture emphasizes individual capabilities, states of being, and overall well-being, focusing on fulfilling potential, fostering self-esteem, and reflecting a culture centered on the individual.

In the Dictionary of Psychology (Chinese version), *mental health* was defined as “a good state in which the individual’s mental state (e.g., general adaptability, soundness of personality) remains normal or at a good level, and in which harmony is maintained within the self (e.g., self-awareness, self-control, self-experience) and between the self and the environment” [24]. According to Zhang and Yang, *mental health* contains objective and subjective components [25]. An individual’s mental health is mainly expressed by the relationship between the individual and others in a group, so it contains social meaning. Hu suggests that *mental health* is about “following one’s heart and not exceeding the rules,” which has both its individual (developmental and autonomous) and social (adaptive and normative) aspects [26]. Yip defines *mental health* as a direction that suggests self-discipline and obedience to social order to maintain inner balance and external harmony with others [27]. Specifically, individuals can maintain this balance and harmony across three levels: personal, interpersonal, and moral/ethical. These definitions underscore Chinese scholars’ emphasis on the social aspects of the individual in conjunction with the proper functioning of mental faculties. They highlight Chinese culture’s focus on harmony, interpersonal relationships, societal connections, and moral/ethical considerations.

In summary, concepts and understandings of mental health are closely tied to culture [28], reflecting that the connotations of mental health defined by different cultural contexts can vary to some extent. Then, how is mental health related to culture? The theory of sociocultural models (TSCM) provides a perspective on the interaction between culture and the individual mind [29].

The primary thesis of the theory of sociocultural models (TSCM) is that the human mind and culture mutually constitute each other. During continued interactions, individuals internalize the social culture into their psychological realities to regulate their actions and interactions. Conversely, community members will externalize the psychological reality through enactment and instantiation, creating new social cultures through social interactions and co-construction with the existing social culture. The dialectical interactions of these two aspects constitute the mechanism of the sociocultural regulation of human actions and the construction of the sociocultural reality [29]. Consequently, social culture dictates varying expectations for mental health standards, while the characteristics associated with mental health are also culturally rooted and reflect social culture. Simultaneously, societal depictions of mentally healthy individuals contribute to the evolution of novel cultural norms in a reciprocal manner.

The Chinese culture has a long history of rich mental health concepts deeply rooted in philosophies such as Confucianism, Buddhism, and Taoism. Confucianism seeks to go into the society(*Rushi*), i.e., “To ordain conscience for Heaven and Earth, to secure life and fortune for the populace, to carry on lost teachings of ancient sages, to build peace for posterity” (Zhang Zai: *Heng Qu Yi Shuo*). When encountering setbacks, Confucianism advocates being adaptable to circumstances and maintaining mental health by being resilient and motivated. Taoism seeks to transcend the world(*Chaoshi*) and advocates “letting go.”When encountering difficulties, people maintain mental health by going with the flow and doing what they should do. The philosophy also underscores the importance of balancing Yin and Yang, enabling individuals to perceive challenges holistically by acknowledging both positive and negative aspects. Buddhism seeks to jump out of the material world(*Chushi*) and advocate “being free of worried thoughts” when encountering difficulties. As Hui Neng(the Sixth Patriarch of Zen) said in the Tan Jing, “Since everything is naught, where can dust gather?” Individuals can cope better with difficulties if they have a mindset that looks down on gains and losses and that everything is nothingness.

Popular anecdotes and proverbs in Chinese culture also dictate criteria for individuals’ mental health. For instance, the “Three Feet of Space” tale narrates an incident from ancient China where the Guo family faced a boundary dispute with their neighbor during house construction. Upon hearing of this issue, patriarch Guo Pu wisely proposed, “Sending letters a thousand miles just for a wall; why not give him three feet?” This led to the Guo family’s compromise, and finally, both families conceded three feet of space from their walls. This narrative underscores the cultural emphasis on fostering interpersonal harmony through mutual accommodation, viewing discordant relationships as signs of poor mental health.

Contemporary scholars have also endeavored to directly integrate key concepts from Chinese traditional culture into psychological counseling and therapy. Yang and his colleagues(2002) [30] created Taoist Cognitive Therapy to facilitate cognitive restructuring in psychologically distressed individuals by directly applying the 32 characteristics of the Taoist principle of health, that is: “Benefit without harm, but not disputing; abstinent contentment with little selfishness and desire; under the knowledge and the place, let gentleness overcome rigidity; recover the original simplicity, let it be.” Liu(2023) posits that “unity of universe and human” in Chinese culture is a core idea of mental health [31]. He pointed out that the psychological phenomenon corresponding to this concept is psychological nothingness. By fusing modern psychotherapy with the concept of “unity of universe and human,” Liu developed the technique of “Moving symptom’s symbol to nothingness” to fulfill the healing role of Chinese culture. These endeavors establish a robust framework for comprehending mental health through the lens of Chinese cultural perspectives.

Over the years, numerous scholars have delved into the attributes of mentally healthy college students. Prominent among these is Wang and Zhang’s widely recognized framework, which outlines eight characteristics drawing from personal experience: understanding and accepting oneself; accepting others and dealing well with them; facing reality squarely and accepting it; loving life and enjoying work; being able to coordinate and control emotions and being in a good state of mind; having a complete and harmonious personality; having normal intelligence; and having age-appropriate mental behavior [32]. However, this work has predominantly focused on psychological cognition, emotion, and intention, with limited consideration of the cultural context, particularly the influence of Chinese culture on mental health.

Subsequently, scholars such as Zeng and Lei, incorporating social, ethical, and moral perspectives, proposed a culturally nuanced framework emphasizing four main traits in mentally healthy college students: positive and controllable emotions, good moral values, comfortable coping with schoolwork, and healthy social interaction [33]. While valuable, this perspective primarily mirrors researchers’ subjective experiences and authority-driven viewpoints. It neglects insights from laypeople, omits identification of the aspects of Chinese culture showcasing characteristics of mentally healthy college students, and lacks differentiation between mentally healthy college students and other demographic groups. Consequently, there is a demand for exploring innovative methodologies to scrutinize the attributes of mentally healthy college students, particularly focusing on characteristics within Chinese culture.

Currently, there are various research paradigms for the study of mental health. Jiang (2004) categorized them and concluded that there are two main principles in evaluating mental health: the majority principle and the elite principle [34]. The majority principle refers to a research paradigm that selects research subjects through large samples and measures whether individuals deviate from the norm through the principle of statistical normal distribution [35]. An example is applying the Chinese version of Symptom Checklist-90 (SCL-90), one of the most often used self-report symptom inventories to measure the mental health of college students, and individuals scoring exceeding the norm were considered abnormal [36].

The elite principle refers to a research paradigm that focuses on elite samples, namely a small number of relatively outstanding individuals in the whole population who are at the tip of one side of the normal distribution, and primarily employs qualitative research methods to derive research findings [35]. For example, Maslow researched some great people in Western history( i.e., self-actualized people) using qualitative research methods such as biographical analysis, depicted 15 characteristics of self-actualized people, that is, “more efficient perception of reality and more comfortable relations with it,” “acceptance (self, others, nature),” “spontaneity; simplicity; naturalness,” “problem centering,” “the quality of detachment; the need for privacy,” “continued freshness of appreciation,” “autonomy; independence of culture and environment; will; active agents,” “the mystic experience: the peak experience,” “gemeinschaftsgefuhl,” “interpersonal relations,” “the democratic character structure,” “discrimination between means and ends, between good and evil,” “philosophical, unhostile sense of humor,” “creativeness,” “resistance to enculturation; the transcendence of any particular culture” [37].

Maslow’s findings profoundly influenced research on mental health definitions, standards, and interventions. While some researchers have embraced the characteristics of self-actualized people as an ideal standard of mental health [38], others have leveraged these characteristics by focusing on exceptional psychological qualities rather than normative behavioral performance [39], and many of these characteristics have been used as ideal indicators of mental health for the promotion of mental health among college students [40]. Additionally, these characteristics and the conditions that promote or inhibit self-actualization are also applied in methods and paths of healthy human development [41]. Furthermore, specific characteristics such as a “philosophical, unhostile sense of humor” have been directly applied by researchers to enhance humor quality among college students facing stress and embarrassment, aiming to uphold their mental well-being [42].

Despite significant value in both theory and practice, Maslow’s study is based on the Western culture and is not aimed at a specific group of college students. Consequently, its direct relevance to enhancing the mental well-being of Chinese college students may be limited, necessitating further investigation into mental health within the framework of Chinese culture. Nonetheless, Maslow’s study of the elite samples of self-actualized people also provides a new research paradigm for mental health research, which has greatly inspired this study.

In the past, most studies on the mental health of college students used quantitative studies based on the majority principle. While some qualitative studies inquiries delved into the characteristics of mentally healthy college students, these studies often focused on specific subgroups like those who experienced being left behind [43] or childhood trauma [44]. A gap exists in the mental health characteristics based on the Chinese culture of college students who are the elite samples, i.e., those who exhibit very good mental health. By utilizing the elite principle paradigm, researchers can gain insights into and depict the mental health characteristics of college students within the context of Chinese culture, with the ultimate aim of delineating the mental health characteristics of college students specific to this cultural framework.

This study will apply the elite principle to examine college students with very good mental health. Through a distinctly Chinese cultural lens, this research aims to delineate what mentally healthy college students look like and what characteristics they show. By focusing on college students’ personal experiences and Chinese culture, this study positions college students as knowledge generators, employing a qualitative research approach to uncover the characteristics of mentally healthy college students. The objective is to achieve a new understanding of college students’ mental health based on Chinese culture and provide a theoretical basis for new mental health standards and a reference for promoting, cultivating, and intervening in college students’ mental health.

In this study, mental health refers to the good psychological state of an individual. College students refer to the group of students who are receiving professional higher education. Chinese culture refers to the culture created by the Chinese over thousands of years of development, from ancient times to the present [45].

## Methods

### Design

**Fig. 1:**
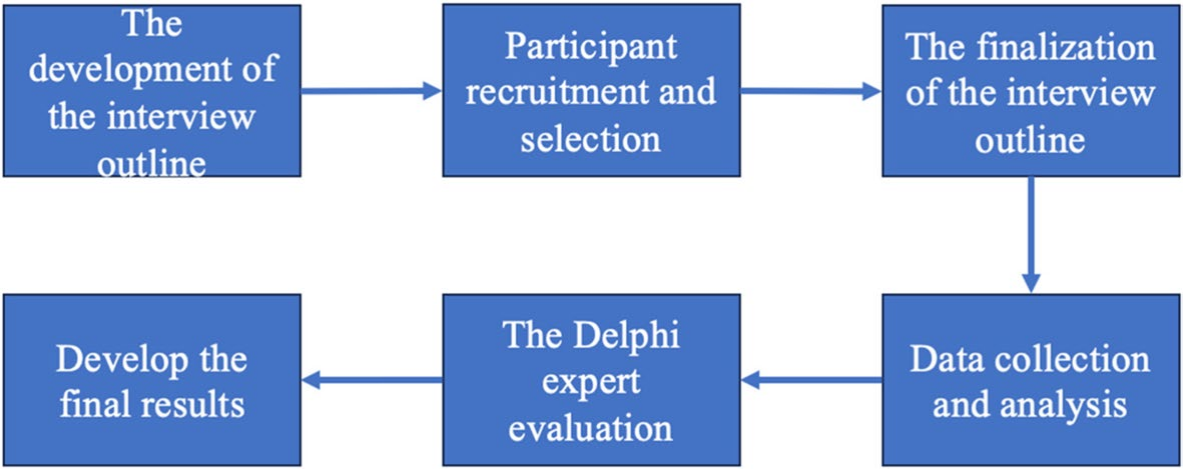
The procedure of the study

The study applied a participatory, exploratory, qualitative design. Qualitative methods are suitable for exploring the meaning of phenomena or life events to the interviewees and their inherent experiences from the subjectivity of the interviewees [46]. It also emphasizes the participants as a generator of knowledge and the acquisition of significant experiences from the participants [47]. Thus, it can help researchers to gain a deeper understanding of community members in a specific cultural-historical context. Moreover, qualitative methods hold particular promise for prioritizing participants’ voices, and they contribute to understanding human interaction with the environment in development and helping researchers build and expand new concepts and theories in specific cultural-historical contexts [48]. This study used semi-structured individual in-depth interviews to explore the characteristics of mentally healthy college students based on Chinese culture. Moreover, the procedure of the study is shown in Fig. 1.

### The development of the interview outline

The qualitative data for this study was collected through semi-structured interviews. Interviews serve as a tool to help reveal and understand participants’ perspectives and experiences. The interview outline for this study was based on the theory of sociocultural models [29], focusing on how the interviewed college students understood Chinese culture and which cultures were internalized as characteristics of mentally healthy college students.

The interview outline in the pre-interview includes questions such as “What do you think is mental health? What do you think a ‘mentally healthy’ college student is like? You can use yourself or your classmates as examples.” “What do you think is Chinese culture? What is your understanding of Chinese culture?” “What do you think is related to college students’ mental health in Chinese culture?” (Appendix 1).

### Participant recruitment and selection

The selection criteria for the participants were: i) undergraduate students enrolled in colleges; ii) having a very good psychological status, with a self-assessment of mental health of 7 or more (out of 10); and iii) self-assessment anxiety/depression scores within the normal range.

The study recruited participants through postings in contact groups and forums among different colleges. Undergraduates who satisfied the selection criteria volunteered to participate in the study. At the time of self-referral, enrolled students rated their mental health with the term “Out of ten, how would you rate your mental health?” as well as filled out self-rated anxiety and depression scales [49, 50].

The reasons for considering selection criteria are as follows. Firstly, the research objective is to identify the mental health characteristics of college students with good mental health. Therefore, following the elite principle and referencing Maslow’s self-actualization research paradigm [37], we have chosen exceptionally mentally healthy college students as elite samples for study. Given that statistical analysis commonly regards the top 27% as the criterion for high-score groups [51, 52], a score of 7 out of 10 indicates high mental health levels. Consequently, the study interviewed college students scoring at least 7 points. Secondly, to eliminate individuals with significant biases in the self-assessment of mental health and those potentially experiencing psychological issues, we utilized scores from self-rating scales for depression and anxiety to exclude possible candidates with underlying psychological disorders.

Eventually, 17 college students who met the criteria were selected for interviews in this study. The selection of participants considered factors that might influence college students to develop different understandings of Chinese cultures, such as upbringing, family environment, and educational experiences. The total number of participants was determined based on thematic saturation, i.e., no significant themes emerged with new respondents [53, 54]. Finally, 17 undergraduate students volunteered to participate in the formal interviews, and the self-reported mental health score of the interviewees was 8.11(SD = 0.90) (out of 10). Among the participants, seven were male, and ten were female. Their ages ranged from 19 to 23 years old (mean age = 20.82, SD = 1.33 years), five interviewees were from Double World-Class Project Universities in China, and 5 were first-year students, two sophomores, eight juniors, and two seniors. Participants came from different regions of China; 4 grew up in north China, 1 in northwest China, 2 in southwest China, 2 in south China, 1 in east China, and 7 in central China; 1 from an ethnic minority. 65% were from urban areas, and 29% had no siblings. Additional information on parents’ education level and occupation is shown in Table 1.

**Table 1:** Basic information of final interviewees

Partici-pant	age	School type	Category of major	Year in university	Region	Nationality	Home-town	The only one child	Education-Father	Occupation-Father	Education-Mother	Occupation-Mother	Mental health score	Interview duration	Word count of raw transcript
M1	19	G	Law	1st	1	Han	U	No	3	Teacher	3	Doctor	9.8	65 min	15,600
M2	23	G	Management	3rd	2	Han	U	Yes	3	Office-holder	2	Office-holder	7	70 min	16,700
M3	20	G	Economics	1st	3	Han	R	No	1	Lorry driver	1	Worker	9	57 min	13,000
M4	21	G	Science	3rd	3	Han	R	No	2	Businessman	2	A professional	9	67 min	11,300
M5	20	G	Economics	1st	3	Han	U	No	2	Office-holder	2	Laid off	7	100 min	24,300
M6	22	D	Law	4th	4	Han	U	No	1	Self-employed	2	Private employment	7	81 min	23,600
M7	21	G	Law	2nd	6	Han	U	No	1	Purchasing agent	1	Sales manager	10	98 min	24,700
F1	22	D	Engineering	3rd	2	Mongol	U	Yes	4	Teacher	3	Office-holder	8	50 min	11,900
F2	20	G	Medicine	3rd	5	Han	U	No	3	Employees of state-owned enterprises	3	Self-employed	8	54 min	9,900
F3	20	G	Education	3rd	3	Han	U	Yes	3	Staff	3	Nurse	8	96 min	23,200
F4	20	G	Economics	1st	3	Han	R	No	1	Farmer	1	Farmer	7	58 min	13,800
F5	20	G	Science	3rd	3	Han	R	No	2	Worker	1	Unemployed	8	70 min	13,700
F6	23	G	Management	3rd	2	Han	R	No	1	Farmer	1	Farmer	8	99 min	26,400
F7	23	D	Medicine	4th	2	Han	U	Yes	4	Teacher	4	Teacher	8	75 min	17,700
F8	21	D	Law	3rd	4	Han	R	No	2	Self-employed	2	Self-employed	8	60 min	13,700
F9	19	G	Education	1st	3	Han	U	No	1	Farmer	1	Farmer	8	83 min	13,200
F10	20	D	Science	2nd	5	Han	U	Yes	2	Accountant	3	Teacher	8	69 min	16,300

Participant: *F* female, interviewees, *M* male participants. School type: *D* Double World-Class Project Universities, *G* General Universities. Region: 1 = Northwest China, 2 = North China,3 = Central China, 4 = Southwest China, 5 = South China;6 = East China. Hometown: *U* = Urban areas, *R* = Rural areas. Level of education:1 = junior high school, 2 = High School, 3 = Specialty or Undergraduate, 4 = Postgraduate

After the interviews, participants were thanked for their participation and contribution and were offered 30 RMB (about 4 dollars) for participating.

### The finalization of the interview outline

Before the formal interviews, three college students (one male and two female) who met the selection criteria were pre-interviewed, and the interview outline was adjusted based on the pre-interviews. Specifically, the researchers adjusted ambiguous expressions. For example, in the pre-interview, the researchers found that if they asked the interviewees, “What do you think is related to college students’ mental health in Chinese culture?” They answered how Chinese culture affects college students’ mental health rather than the characteristics of mentally healthy college students based on Chinese culture. Therefore, we adjusted the question to “What a ‘mentally healthy’ college student is like based on Chinese culture? You can take yourself or your classmates as an example” to obtain the characteristics of mental health that reflect Chinese culture. A formal interview outline was eventually formed (Appendix 2).

### Data collection and analysis

The qualitative data was collected through in-depth personal interviews with eligible college students. Each interview lasted between 50- 100 min and was conducted by the researcher (MG), who possessed a doctoral background in psychology, had received training in qualitative research methods, and had three years of experience working in mental health education in universities. All participants signed informed consent forms prior to the interviews. In total, 1252 min of interviews were conducted with 17 participants, which were then manually transcribed by MG, resulting in 289,000 words of interview transcripts.

To accurately ascertain the true meaning expressed by the participants, this study employed manual analysis within the research team to code and analyze the interview transcripts word by word and sentence by sentence. Under the guidance of XJ (a clinical and counseling psychology professor), the research team completed all data analysis work. In addition to MG and WW, the team members included two doctoral students who are also full-time university psychological counselors and two master’s students specializing in mental health education.

The data analysis was conducted using thematic analysis [55]. The steps are as follows: first, the researcher transcribed each of the digitally recorded interviews, immersed within the data, and repeatedly read through the 289,000-word interview transcripts. Second, researchers identified meaningful texts and created open codes. Each meaningful sentence was marked with a “code number,” totaling 1,889. The study used “F” to represent female interviewees and “M” for male participants. The first number represents the interview orders of interviewees; the second number represents the order of the meaningful statements in the interview. For example, “M5-40” represents the 40th word, sentence, or paragraph spoken by the fifth male interviewee. Third, after contemplating the open codes repeatedly, 168 manifestations of mentally healthy college students were derived through initial coding. These manifestations were then summarized to establish 18 characteristics of psychologically healthy university students via focused coding. Subsequently, these 18 characteristics were further classified through core coding to derive five main themes. Fourth, we checked the themes and adjusted their structure until they met internal homogeneity and external heterogeneity criteria. Fifth, we defined and named the themes; 18 characteristics were obtained and coded into five themes.

### The Delphi expert evaluation

Subsequently, three experts were invited to assess the appropriateness of naming, defining, and classifying the identified 18 characteristics and five themes above. These experts are professors in clinical and counseling psychology from institutions such as Beijing University of Chinese Medicine, with in-depth research in Chinese culture and mental health. They have published numerous related monographs and academic papers, such as “When Psychological Counseling Meets Traditional Culture” and “Mind Operations in Meditation.”

The evaluation comprised two rounds. The first round involved a focused group interview where the three experts individually reviewed each theme, characteristic, and original interview data, offering suggestions for revision. They generally approved of the theme divisions and most characteristics, with two main modifications: 1) the integration and categorization of specific characteristics, such as the initial characteristic “Having a pleasant disposition,” which was deemed by experts to contribute to a comfortable interpersonal state and thus was incorporated into “Interpersonal harmony and comfort.” 2) Adjustments to specific nomenclature, such as refining “Showing filial respect to parents” to “Showing filial respect to parents appropriately” to better emphasize the nuance of the characteristic.

The revised results were resent to the three experts for a second round of evaluation, leading to a consensus with no further modifications suggested, thus finalizing the research findings.

### The trustworthiness of the data

Trustworthiness was achieved in several ways.

First, to minimize personal biases to the greatest extent possible, the researchers continuously reflect at each stage of the research project, remaining attentive to the influence of their own experiences and biases throughout all research and analysis phases. For instance, MG utilized a reflective journal [56] to document personal perspectives after each interview, consistently reminding herself to avoid preconceived notions.

Second, the selection of participants considered factors that might influence college students to develop different understandings of Chinese cultures to ensure the diversity of the participants. And, the total number of participants was determined based on thematic saturation [53, 54]. In this study, after interviewing the F8(the 14th interviewee), no new significant themes emerged. Then, three more interviews were conducted (F9, F10, M7), and no significant themes emerged with the new respondents.

Third, the research performed investigator triangulation [57]. Independent researchers completed comparative analyses of individual findings, organized regular research team meetings to compare the analyses, and identified relevant themes. Moreover, XJ frequently reviewed interviews conducted by MG, her reactions to interviews, and the formulation of results. All the researchers discussed the coding and the corresponding original text until a consensus was obtained to bolster the study’s credibility and dependability.

Fourth, external audits are conducted to foster the accuracy or validity of a research study [57]. The research invited three experts above who have made achievements in Chinese culture and mental health to assess the appropriateness of naming, defining, and classifying the characteristics and themes in order to enhance the reliability of research findings.

## Results

### College students’ understanding of Chinese culture

The interviewees’ understanding of Chinese culture was focused on the following four main aspects, and the participant’s identifier follows quotations.

Firstly, Chinese culture is undoubtedly distinct from other countries. For example, F1 believes that “Chinese culture is not just some fixed dynasties in history, or language, or what some literati or educators or some people said, it refers to some patterns of behavior or some ideas that distinguish people from other countries” (F1-66) and is unique to China (M3-110).

Secondly, Chinese culture includes both traditional and modern Chinese new cultures (e.g., revolutionary spirit, M2-95, M4-151, M7-85). Moreover, it is argued that Chinese culture is the essence of what has been left behind through history, including all aspects that have been handed down from ancient times to the present (M1-99; M5-128), and that it is a continuous transmission (F2-72, F4-92, F5-170, F7-137; F9-181; M6-132) and a fusion of the old and the new (F7-142). Chinese culture is implicitly formed and constantly influences and permeates everyone or their lives (F3-134; F7-138; M1-102; M3-111).

Thirdly, Chinese culture is a macro concept, encompassing both intangible and physical aspects. Intangible aspects include thoughts, spirits, and qualities (M2-95, M4-151, M5-131). The physical component includes not only literary works such as poetry (as perceived by all respondents) but also Chinese language and writing (Chinese characters, F2-75; oracle bone inscriptions, F9-184; calligraphy, F2-77, F5-170, F8-94), architecture (F3-148; F10-98), costume (F3-141; F10-101), and folkloric performances (drama, F2 -74; shadow puppets, F5-168; martial arts, F7-141), gastronomy (M5-132), art (painting, F8-93; paper-cutting, M1-100, M2-96, F5-169; china, M2-97; F2-75), traditional festivals and customs (M3-107; F3-138; F5-166; F7-140; F0-97. M7-87) and many others.

Fourthly, some important historical and modern figures mainly reflect Chinese culture’s ideological and spiritual aspects. For example, the famous statesman and literary figure Wen Tianxiang of the late Southern Song dynasty, whose poems “Everyone must die; let me but leave a loyal heart shining in the pages of history books” showed the interviewees his righteousness (M4-122), resilient, his moral integrity (F6-77), and his fearlessness in dedicating his life to his country (M2-72). There is also Zhou Enlai’s ambitious pursuit of “Reading for the rise of China” (M4-62), Mao Zedong’s sense of family and country and the importance he attached to learning (M5-43), and Qian Xuesen’s strict demands on himself during his research (M4-126). The interviewees also made many references to literary figures, such as Li Bai, a poet of the Tang dynasty, whom several interviewees mentioned for his free and ease in the face of frustration (M2-92, M6-30), and his ability to show his spontaneous side in life and learn things spontaneously(M5-54). As well as the ambition of Du Fu showed in his poem “When you are standing on the peak, you are on top of the world” (M5-36), and his sense of responsibility (M1-91, F3-56) reflected in his other poem, “To Emperor Yao and Shun, and to make the customs simple again” (M1-91, F3-56). They also talked about Su Shi’s open-mindedness (F8-79; M6-9) and cheerfulness (M5-29) in the face of adversity, who is a famous poet, calligrapher, gourmet, and hydraulic expert in the Northern Song Dynasty; and also the inner peace(M6-15) and indifference (F3-53) of Tao Yuanming (a famous idyllic poet in the Eastern Jin Dynasty) from his poem “I pick fence side asters at will; carefree I see the southern hill,” and so on.

In addition, the spirit of Chinese culture is also reflected in some allusions and some historical events in ancient and modern times, for example, “Mencius’ mother moves her home three times to better her son’s education” (F1-60), “Che Yin makes use of the light of fireflies or the reflected light by the snow to study” and “Kuang Heng dug a small hole on the wall in order to get some light from the neighbor’s house to read books” (F1-61; F8-34). These allusions convey the importance of studying hard even when conditions are limited. Also, the revolutionary spirit of the May Fourth Movement shows that young people are not afraid of sacrifice (M4-29), and the New Democratic and Industrial Revolution embodied the unity of the Chinese people (M7-91).

### Characteristics of mentally healthy college students based on Chinese culture

There are eighteen characteristics of mentally healthy college students based on participants’ understanding of Chinese culture as described above, which is coded into five core themes: (1) value pursuit, (2) life attitude, (3) interpersonal ideal, (4) behavior ability, and (5) self-cultivation. It can be seen that the vast majority of the mental health characteristics reflect traditional Chinese culture, which is constantly being passed down and changed, with the remainder reflecting the influence of modern Chinese culture. The five themes and corresponding characteristics are shown in Table 2. The results are presented below, and the participant’s identifier follows quotations.

**Table 2:** Characteristics of Mentally healthy college students based on Chinese culture

Theme	Characteristics			
Value Pursuit	① Loving their motherland and identifying with their culture	② Having a sense of responsibility and mission and being willing to dedicate oneself to the country at any time	③ Daring to criticize, explore, and innovate	
Life Attitude	① Loving life and being positive	② Staying positive and having the ability and quality to cope with hardships	③ Being flexible and dialectical	④Being inclusive and broad-minded
Interpersonal Ideal	① Being benevolent and kind	② Interpersonal harmony and comfort	③ Having a soul mate	④ Showing filial respect to parents appropriately
Behavior Ability	① Adapting to the environment	② Studying diligently and learning well	③ Being emotionally appropriate and can regulate emotions	
Self-cultivation	① Having an objective, positive perception of oneself and can accept one’s mediocrity	② Being confident and also modest	③ Focusing on introspection and contemplation to align with the sages	④ Possessing good qualities advocated by Confucianism, Buddhism, and Taoism coexist harmoniously

#### Value pursuit

Value pursuit refers to an individual’s understanding and practice of life ideals and beliefs after integrating social consciousness, such as worldview, life view, and values. Participants described that mentally healthy college students based on Chinese culture have strong beliefs and goal pursuits of contributing to the motherland. They exhibit profound loyalty towards their motherland, viewing its service as their sacred duty, and are steadfast in their resolve to contribute through bold exploration, even in the face of daunting challenges or the prospect of personal sacrifice. This theme directly reflects the Chinese Confucian culture of “Self-cultivation is the starting point of several steps moving outward. The next step is managing family affairs, followed by governing the state. The final step is moving to provide peace and sound governance to all under heaven” and “To be the first in the country to worry about the affairs of the state and the last to enjoy oneself.” The following three subthemes were identified regarding students’ value pursuit.

##### (1) Loving their motherland and identifying with their culture

First and foremost, mentally healthy college students love their country and are firmly convinced that they want to identify with it. Twelve interviewees emphasize that mentally healthy college students should embody love for their country, cultural identification, and a profound sense of belonging and national pride. On the one hand, they are patriotic and loyal to their motherland and have high moral characters. For example, one participant said, “*like the patriotism in Yue Fei (a famous military man, strategist, calligrapher, poet, and national hero in Chinese history, and was the first of the Four generals rebuilding the Song dynasty). His patriotism and loyalty are also what a mentally healthy college student should have*” (#M6-54).

On the other hand, they identify with the country, nation, and culture from the heart and are proud of the motherland. Another participant said, “*Mentally healthy college students should have a real sense of cultural identity. Furthermore, a Chinese should identify with the traditional Chinese culture ……*” (#F3-110).

##### (2) Having a sense of responsibility and mission and being willing to dedicate oneself to the country at any time

In addition, mentally healthy college students have a firm sense of mission and responsibility to the motherland. Ten interviewees assert that mentally healthy college students should exhibit a sense of national responsibility, ambitious aspirations, and a readiness to devote themselves to their homeland wholeheartedly. Mentally healthy college students should have ambitious ambitions. As M1-75 said: “*‘To ordain conscience for Heaven and Earth, to secure life and fortune for the populace, to carry on lost teachings of ancient sages, to build peace for posterity’ (Zhang Zai: Heng Qu Yi Shuo), which can also reflect the looks of a mentally healthy college student.*”

The most important thing is to be willing to contribute to their motherland, even at the expense of oneself. Another participant said, “*Mentally healthy college students do not think about personal gains and losses too much but put their country and nation before themselves, ……, ‘Death is not my concern should it benefit the country. How can I pick and choose for my loss or gains?’ (Lin Zexu) ……*” (#M7-22).

##### (3) Daring to criticize, explore, and innovate

At the same time, mentally healthy college students have the quest and conviction to keep climbing to the top. Sixteen interviewees believe that mentally healthy college students are enterprising, daring to criticize, explore, and innovate to contribute to their country’s development. Mentally healthy college students are active, enterprising, and have goals and plans. One participant said, “*I think mentally healthy college students should have goals and plans for themselves*” (#M6-3). They also have critical thinking and exploratory spirit and will keep innovating. As F7 said, “*If you are a mentally healthy college student, you also need some innovative spirit to break through ……*” (#F7-59). Also, they are willing to explore and contribute to the country’s development, as M4 said: “*Mentally healthy college students should be like Qian Xuesen (also known as Tsien Hsue-she), who has a strong spirit of patriotism. He devoted himself to scientific research, and after countless attempts and explorations, he finally launched the first atomic bomb for China ……*” (#M4-124).

#### Life Attitude

Life attitude is an individual’s understanding and reaction to things that happen in daily life. Participants highlighted that maintaining a positive, optimistic, dialectical, and open-minded stance towards setbacks and challenges is a key characteristic of mentally healthy college students. This theme directly reflects Chinese culture: “Someday, with my sail piercing the clouds, I will mount the wind, break the waves, and traverse the vast, rolling sea.” and “It is blessed to suffer losses.” The following four subthemes regarding students’ life attitudes were identified.

##### (1) Loving life and being positive

Mentally healthy college students hold positive attitudes about life. Fourteen interviewees believe that mentally healthy college students exhibit optimistic attitudes toward life. Mentally healthy college students approach life optimistically, viewing it as brimming with hope. As F9 mentioned, “*I think I am mentally healthy because I am quite positive and optimistic about life, and I will face it positively even if there are some bad things*” (#F9-149). Moreover, they love life and experience life from their heart, “*I think mentally healthy college students can live a good life. Particularly, they can still maintain a love for life, have something they want to do, have the energy to fight or to live.*” (#M2-2). They always think life is full of meaning. As F1 said, “*I think some of the cases (of mental ill health) are because they have lost hope in life and do not want to do anything*” (#F1-47).

##### (2) Staying positive and having the ability and quality to cope with hardships

Mentally healthy college students possess a positive attitude towards suffering and setbacks. All interviewees believe that mentally healthy college students have a positive view and the qualities of coping with suffering when facing life difficulties. They will not shy away from adversity; instead, they proactively address issues, surmount obstacles, and manage them with composure. When facing difficulties or setbacks, mentally healthy college students maintain constructive beliefs. As one participant said: “*‘Just as heaven keeps moving forward vigorously, a man of virtue should strive continuously to strengthen himself’ (The Change of Book). And ‘When Heaven intends to confer a great responsibility upon a person, it first visits his mind and will with suffering, toils his sinews and bones, subjects his body to hunger, exposes him to poverty and confounds his projects. Through this, his mind is stimulated, his nature strengthened, and his inadequacies repaired’ (Mencius). A mentally healthy college student should be like as described in these statements.*” (#F9-25).

They also exhibit the qualities to cope with hardships, such as striving continuously to strengthen themselves, being indomitable, resilient, enterprising, and so on. “*I think indomitable also reflects the self-control mentioned earlier, that is, they will not give up even after experiencing more difficulties*” (#M4-136).

Furthermore, they can analyze and resolve problems amid adversity and challenges, effectively overcoming them. “*For a long time, when my friends and I encounter setbacks, crises, or challenges, I always use this phrase to encourage myself and others to handle it calmly, ‘to be unchanged in front of the collapse of the mountain Tai, and to face danger without being surprised when it suddenly comes in front of you.’*” (#M7-6).

##### (3) Being flexible and dialectical

Mentally healthy college students have a dialectical attitude towards life. Ten interviewees noted that mentally healthy college students demonstrate critical thinking skills by approaching situations objectively, comprehensively, and dialectically. These dialectical concepts, attitudes, and behaviors when facing negative things in life are also characteristics of mentally healthy college students. One participant said, “*Mentally healthy college students should be as objective and comprehensive as possible when dealing with things*” (#F3-118). They do not dwell on the present and have a positive attitude toward the future, “*There are plenty of fish in the sea. Do not miss the whole forest because of a tree. Even if you are sad about a breakup, do not cling to the past, but try to live a new life*” (#M7-12).

Furthermore, they think dialectically and believe that all sufferings have its reward. As F1 said: “*A saying goes that ‘Someday This Pain Will Be Useful to You,’ which means that it is not always bad to suffer Loss; think long term. For example, one may sometimes feel that their interests are being threatened in interpersonal relationships. However, if they are particularly concerned about this, it will make them uncomfortable, while if they are generous or forgiving, their heart will become more open*” (#F1-24).

##### (4) Being inclusive and broad-minded

Mentally healthy college students have an open-minded attitude toward life. Sixteen interviewees believe mentally healthy college students are tolerant, broad-minded, and open-minded. Both for themselves and others, mentally healthy college students hold tolerant attitudes. A participant said, “*I may lack a little tolerance for others because I am always strict with myself, so I may sometimes be strict with others. So, from this point of view, I think my mental health level needs to be further improved*” (#M2-79). They are broad-minded (“*Be magnanimous, as the saying goes, ‘A prime minister’s mind should be broad enough for poling a boat,’ which is a sign of college students’ mental health, advising people to look at whatever things a little more openly*”, #F6-34).

Moreover, even in the face of life’s misfortunes, they are also very liberal and open-minded, able to accept them openly. As M6 mentioned, “*One should also have positive and healthy perceptions. Su Shi, a famous poet, calligrapher, gourmet, and hydraulic expert in the Northern Song Dynasty, openly accepted the fact that he was deprived of his official position. Instead of being depressed daily, he lived an easy and interesting life, free and relaxed*” (#M6-9).

#### Interpersonal ideal

Interpersonal ideals refer to the pursuit and aspiration of individuals to achieve the best in interpersonal communication and good relationships. According to these interviewees, the characteristics of mentally healthy college students can be divided into general and specific interpersonal relationships. Regarding general interpersonal relationships, mentally healthy college students are friendly and kind, and their interactions with others are harmonious and comfortable. When navigating specific relationships like those with parents, they are filial but have rational thinking; in terms of friendship and romantic partnerships, they pursue ideal and pure relationships. This theme is a direct reflection of Chinese culture: “benevolence,” “harmony is precious,” “The relations between men of virtue are plain like water,” “filial piety,” and so on. The following four subthemes were identified regarding students’ interpersonal ideals.

##### 1） Being benevolent and kind

Mentally healthy college students are benevolent and kind in their interactions with others. Thirteen interviewees believe mentally healthy college students are kind-hearted, compassionate, sincere, caring, and helping others without discrimination. Mentally healthy college students are benevolent and have compassion for others; as M7 mentioned, “*When I met beggars on the road, …… whether they are pretending or be, I am always willing to give them some money……*” (#M7-54). They are kind-hearted (“*I think a person should be at least kind-hearted; he may have that kind of empathy inside, have that kind of emotion for either other people or animals, ……, and have a softer heart, which also reflects the mental health of college students*,” #F6-45). They treat people gently and friendly (“*Laozi and Confucius look gentler than others, I feel that this characteristic in them also indicates the mental health of college students*,” #M3-73).

Furthermore, they are helpful and kind to others. As one participant said, “*Imagine this: You’re in a crowd, and a bike tumbles to the ground. Everyone is looking around, unsure of what happened. Now, you’re caught in a bind: Should you lend a hand or stay back to avoid being wrongly accused? Despite the chance of misunderstanding, I feel it’s crucial to step up and help. Ignoring the situation just doesn’t sit right with me—it goes against everything I believe in.*” (#F5-161).

##### 2） Interpersonal harmony and comfort

Mentally healthy college students have a harmonious and comfortable interpersonal state. All interviewees agree that mentally healthy college students exhibit pleasant character and interpersonal adeptness, adhere to fundamental Chinese cultural values, and maintain a more harmonious and comfortable relational environment compared to their peers. Mentally healthy college students experience interpersonal harmony and comfort; one interviewee said, “*A mentally healthy college student has better interpersonal relationships, ……and has a comfortable social state*” (#F1-17). In interpersonal interaction, they prioritize harmony (“*I quite agree with the saying ‘Peace is of paramount importance. Since we are studying together, it is important to take care of each other and try to understand each other*”, #M3-49). Besides, they have good interpersonal interactions (“*ones’ mental health, I think, also shows more in whether they can deal with interpersonal relationships with people around them, …… whatever kind of people may meet, they can deal with the relationship well*”, #F6-9).

Moreover, they appreciate others (“*If other people have gained a certain amount of academic achievement, …… if he is (mentally) healthy, he may be happy for others’ success, achievement*”, #M7-33). Also, they can resolve conflicts or contradictions in interpersonal relationships (“*There is no perfect person; for example, if they cause harm to others, they can recognize their mistakes and apologize timely and honestly*,” #M6-101).

Furthermore, they follow many guidelines to create a harmonious and comfortable interpersonal state. As F3 mentioned, “*I think, when it comes to some unimportant things, it is important not to bother others like that…… one should have the sense of proportion*” (#F3-39).

##### 3） Having a soul mate

Mentally healthy college students seek to have a soul mate in specific friendships or romantic partnerships. Nine interviewees suggest that mentally healthy college students possess the ability and quality to communicate and empathize with others on a deep spiritual level and form corresponding friendships or romantic relationships. Whether in friendship or romantic relationships, mentally healthy college students have the correct attitude toward interaction, as F8 said, “*For example, Zeng Gong and Wang Anshi (both politicians of the Northern Song Dynasty), …… They become good friends for life not based on interests, but on their appreciation of each other, and the same values, which I think mental health of college students should always be*” (#F8-67).

They emphasize the spiritual level of communication more than pursuing each other’s company. They have a more high-quality and pure relationship, in friendship or romantic relationships. As F6 said: “*‘The friendship of a noble person is as pure as water.’ (Chuang-Tzu). Put simply, relationships should be genuine and straightforward, free from fame-seeking or ulterior motives; Just like the story of Boya and Ziqi, mentally healthy students might find a companion who truly gets them, connecting on a spiritual and empathetic level……*” (#F6-38). It is the same with romantic relationships, as M6 mentioned, “*When you read the poem of Su Shi, for example, ‘Ten years parted, one living, one dead; Not thinking; Yet never forgetting; A thousand Li from her lonely grave; I have nowhere to tell my grief……’ The affection between him and his wife is so deep that it is enviable*” (#M6-42).

##### 4） Showing filial respect to parents appropriately

Mentally healthy college students have rational conceptions of filial piety towards their parents and appropriate, respectful behavior. Eleven interviewees believe mentally healthy college students are filial and rational in their interactions with their parents. Mentally healthy college students show filial piety to their parents appropriately. On the one hand, they practice filial piety by accompanying their parents, communicating more with them, caring for them, repaying them, and so on. As F5 mentioned, “*‘Our bodies—to every hair and bit of skin—are received by us from our parents’ (Xiao Jing). Mentally healthy college students are grateful and respectful, often care for their parents, and spend more time with them*” (#F5-109).

On the other hand, they also have rational thinking rather than unprincipled obedience regarding filial piety’s “cognition” aspect. As one participant said, “*Not just any kind of filial piety, that is, you should have your thinking and judgment……*” (#F3-105). Another participant said, “*Proper filial piety is an aspect of college students’ mental health, not that they are obedient to their parents. When they disagree with parents, they can communicate more with parents and let themselves be understood*” (#M5-102).

#### Behavior ability

Behavior ability refers to the ability of an individual to behave appropriately. According to these interviewees, mentally healthy college students have a variety of behavioral abilities, such as adapting to different environments, learning well, and regulating their emotions. This theme directly reflects the Chinese culture: “Those who obey heaven survive, and those who defy heaven perish,” “learn without thinking is reckless, think without learning is dangerous,” and “When joy, anger, sorrow, and happiness are not yet expressed as a response to other things, they are in a state of balance. When they are expressed in words and deeds by the rites, harmony is achieved. “The following three subthemes were identified regarding students’ behavior ability.

##### (1) Adapting to the environment

Mentally healthy college students can adapt to the environment. Seven interviewees believe that mentally healthy college students can adapt to different environments. Adaptability is reflected on the one hand in the interpersonal aspects (“*There is also the adaptation to the university environment. Mentally healthy college students can integrate into groups and clubs, and actively participate in club activities*”, #F2-16). Also, they can adapt to different environments (“*I think social adaptability is quite important…… I went to work part-time this summer, but I feel that I have just been exposed to it*”, #F9-10). Moreover, they also show adaptability to adversity (“*I think mentally healthy college students also can adapt to adversity……*”, #M5-70).

##### (2) Studying diligently and learning well

Mentally healthy college students can learn well. Thirteen interviewees suggest that mentally healthy college students exhibit a positive learning attitude, take ownership of their learning, maintain a continuous learning process, and demonstrate good study habits. They learn earnestly and diligently and have good learning attitudes (“*College students with good mental health will keep learning, have the initiative to learn, down-to-earth. Moreover, if they work by fits and starts (Cao Xueqin: The Dream of Red Mansions), there will not be a good result*”, #F5-64).

They also actively take responsibility for learning. As F10 said, “*Responsibility is fundamental. The primary task for students is studying. One should stay in one’s lane*” (#F10-83). Besides, they are good at learning (“*I think Lu Xun, who gave up medicine to pursue literature, …… has a powerful ability to learn*”, #F9-71). In addition, they study diligently and accumulate knowledge. As M2 mentioned, “*Since I have to prepare for the entrance examination, I have to memorize words and take lessons every day. That is, ‘But unless you pile up little steps, you can never journey a thousand li; unless you pile up tiny streams, you can never make a river or a sea.’ (Hsun-Tzu: Encouraging Learning), …… I realized that what I do daily is important*”, #M2-93).

##### (3) Being emotionally appropriate and can regulate emotions

Mentally healthy college students can regulate and manage their emotions. Nine interviewees posit that mentally healthy students display emotional appropriateness and stability, promptly and effectively managing their emotions. Emotions are often regarded as the signal light of mental health. Thus, mentally healthy college students are emotionally appropriate and relatively stable, “*A mentally healthy college student should be emotionally stable, …… ‘The master was mild, and yet dignified; majestic, and yet not fierce; respectful, and yet easy’ (The Analects). One should have a suitable emotion in which state*” (#F3-78).

Moreover, when encountering adverse events, they have the ability to regulate their emotions. As one participant mentioned, “*A mentally healthy college student can control his emotions and regulate his emotions*” (#F6-1). At the same time, they can adjust themselves in appropriate and healthy ways in time, “*when he meets some bad things, he can just communicate with others, exercise…… instead of drinking or even hurting himself*” (#F8-10).

#### Self-cultivation

Self-cultivation refers to the inner quality or state an individual constantly improves or achieves through long-term efforts and cultivation. According to the interviewees, mentally healthy college students advocate the continuous improvement of self-cultivation. They try to possess many excellent qualities of Confucianism, Buddhism, and Taoism and perfect them daily by having clear and objective self-knowledge and constantly reflecting on themselves to improve their cultivation. This is a direct reflection of the Chinese culture of “no end to learning” and “Seeing the virtuous and thinking of the wise, seeing the unwise and introspecting”, and so on. The following four subthemes regarding students’ self-cultivation were identified.

##### (1) Having an objective, positive perception of oneself and can accept one’s mediocrity

The constant improvement of mentally healthy college students’ self-cultivation first requires a clear perception of oneself. Eleven interviewees believe mentally healthy college students have a positive, comprehensive, and clear understanding of themselves. They know their strengths and weaknesses and can accept their mediocre and weak sides, “*For example, an Olympic weightlifter, he can only lift 50 pounds, but he had to go lift 100 pounds…… A mentally healthy person should clearly understand themselves and do according to one’s abilities…*”, #F8-33). They also have a positive view of themselves, “*‘All things in their being are good for something’ (Li Bai: Invitation to Wine); one should not think too lightly of themselves when disillusioned. They can certainly play their usefulness in life, cannot improperly belittle oneself*” (#F9-35). Furthermore, they can also accept their mediocrity and weakness, “*I think there is also a significant point, which is to accept their mediocrity gradually……*” (#F1-8).

##### (2) Being confident and also modest

The constant improvement of mentally healthy college students’ self-cultivation also requires an objective perception of oneself. Thirteen interviewees believe that mentally healthy college students are confident and able to stick to what they believe is correct while also being modest. According to a participant, mentally healthy college students believe in themselves, “*This point of believing in oneself in Qian Xuesen is probably also what a mentally healthy college student should have……*”, #M4-128). They are assertive and can stand firm on their ideas (“*When faced with two choices, mentally healthy college students listen to others’ opinions and at the same time stick to their ow*n,” #F4-77). At the same time, they are also modest (“*A saying goes that, ‘Modesty helps one go forward, whereas conceit makes one lag.’ In my opinion, mentally healthy students may not be so proud of themselves……”, #F5-36).* Furthermore, they are not overly confident or modest *(“Both confidence and modesty in a mentally healthy college student are appropriate and balanced, that is, I think it is necessary to be confident but also modest……,*” #F7-109).

##### (3) Focusing on introspection and contemplation to align with the sages

Mentally healthy college students improve themselves through constant introspection. Ten interviewees believe mentally healthy college students focus on introspection and are strict with themselves. They constantly check the gaps to seek progress and expand their horizon. Specifically, mentally healthy college students often reflect on themselves (“*‘I daily examine myself on three points……’ (The Analects) which I think reflects the mental health of college students, that is, whether you are doing your best in the team……*”, F2-35). They are also strict with themselves, “*As the sayings go, ‘You cannot expect a better world without cleaning your room first,’ although Du Fu (a famous poet of the Tang Dynasty) is said to be very talented, if one cannot do small things well, like cleaning the house, he can do nothing else well*” (#M5-52).

Moreover, they make constant progress and look to the virtuous, “*‘When you see a person of virtue and capability, you should think of emulating and equaling the person; when you see a person of low caliber, you should reflect on your weak points’ (The Analects). Mentally healthy people also constantly learn from the strengths of others and reflect on their weaknesses*” (#M2-34).

##### (4) Possessing good qualities advocated by Confucianism, Buddhism, and Taoism, which coexist harmoniously

The highest level of self-cultivation for mentally healthy college students is to possess many good qualities of Confucianism, Buddhism, and Taoism, which together become the characteristics of mentally healthy college students. Sixteen interviewees suggest that mentally healthy college students exhibit strong moral characteristics and virtues from Confucianism, Buddhism, and Taoism, all coexisting harmoniously. Mentally healthy college students have the excellent qualities of Taoism, such as being calm and bland, indifferent to fame and fortune, and peaceful and happy. As the participants said, “*This sense of ordinariness, which I think may also be a necessity for mental health……*” (#F7- 34); “*Mentally healthy college students are calm and relaxed, take the rough with the smooth; they have confidence in themselves and take it easy*” (#M7-35).

Moreover, they have the excellent virtues of Confucianism, such as benevolence, righteousness, rites, wisdom, and good faith. As F3 said, “*Mentally healthy college students must be good in these virtues, like ‘loyalty, filial piety, rites, wisdom, good faith, and courage’……*” (#F3-90). Another participant mentioned, “*After comparing so many fictional characters, it is hard for me to use words to describe him (Qiao Feng), …… very filial and loyal, very righteous, …… doing things very fairly, … …*”, #M6-59).

Besides, they also obtain the main qualities of Buddhism, such as gratitude and kindness (“*‘Moral character can be built by accumulating goodness’ (Hsun-Tzu: Encouraging Learning). A mentally healthy college student does good deeds, such as attending activities as a volunteer……*” #F2-30). As F9 said, “*Also, mentally healthy college students often remember others’ kindness and are grateful, and then be nice to others, as the saying goes, ‘You throw a peach to me, I give you a white jade for friendship.’ (The Book of Songs)*”, #F9 -112).

## Discussion

The study identified five themes and 18 characteristics of mentally healthy college students within Chinese culture. These characteristics are deeply rooted in Chinese traditions, highlighting yin-yang balance and moral cultivation. They related closely to college students’ identity, learning stage, and age. Contrasting with characteristics of other cultural backgrounds, they showcase the impact of Chinese culture on college students, validating and expanding the theory of sociocultural models.

### Comparison with previous studies

Firstly, compared to existing research on the characteristics of mentally healthy college students, this study presents novel findings and unique insights. Consistent with other related studies rooted in Chinese culture, both this study and previous research accentuate that the characteristics of mentally healthy college students encompass facets such as self-awareness, interpersonal relationships, emotional regulation, and positive learning traits. For instance, Wang (1992) posited that mentally healthy college students exhibit characteristics focusing on self-awareness, interpersonal adeptness, and emotional regulation [32]. Similarly, Zeng (2021) described the characteristics of mentally healthy college students, highlighting their emotional state, academic performance, and interpersonal skills [33].

Some characteristics revealed in our study diverge from those proposed in prior research concerning their specific connotations. Taking emotional regulation as an example, the research of Zeng (2021) and Wang (1992) primarily emphasized affirming positive emotions. They depicted mentally healthy college students as “positively emotional and controllable” or “possess the capacity to coordinate and manage emotions effectively, sustaining a positive mood.” In contrast, the characteristic identified in this study of “being emotionally appropriate and can regulate emotions” not only encompasses positive emotions but also includes negative feelings, emphasizing the timely and moderate expression of both. This directly reflects the Confucian concept of “Zhongyong” (doctrine of the mean) in Chinese culture, which advocates for moderation in all things, whether positive or negative. Therefore, it is evident that college students’ mental health is closely intertwined with the concept of moderation. Individuals can achieve mental health in various aspects by expressing emotions moderately, whether positive or negative.

Furthermore, this study has identified characteristics not previously mentioned by Chinese scholars, such as “showing filial respect to parents appropriately.” Filial piety is a unique social behavior within Chinese culture, embodying a comprehensive and intricate ethical framework [58]. Chinese society dramatically emphasizes family values, where treating parents well and acknowledging their upbringing is paramount. Therefore, if one is not filial, one cannot be said to be mentally healthy. However, with the evolution of societal norms, the essence of filial piety has transformed. Recent research reveals that contemporary society no longer adheres to traditional interpretations of filial piety solely through obedience to parents [59]. This shift signifies that mentally healthy college students now approach filial piety differently, manifesting altered perspectives, attitudes, and behaviors toward this concept. In ancient China, departing from one’s hometown to pursue education and personal growth was discouraged, as staying by one’s parents’ side was deemed the epitome of filial piety. As Confucius stated, “While the father and mother are living, do not wander afar” (*The Analects*). However, today, individuals are encouraged to venture afar to contribute meaningfully to their country and society [60]. As a result, modern manifestations of filial piety among mentally healthy college students involve not just reverence, care, and support for their parents but also underscore the significance of preserving autonomy and independence while fulfilling their familial duties.

Secondly, upon comparing our findings with research from other cultural backgrounds, it becomes apparent that our results diverge significantly from those of Western culture but align closely with research outcomes from Africa and Asia.

In the West, the understanding of mental health emphasizes enhancing personal belonging, satisfaction, and well-being, which is very different from Chinese culture, which emphasizes self-sacrifice and self-elimination [61]. Although this study was conducted in a qualitative study of a group of college students in very good mental health, a research perspective similar to Maslow’s research on self-actualizers, there were significant differences in the specific characteristics of these healthy individuals in different cultures. In particular, this study did not address the characteristics of self-actualizers noted by Maslow, such as “the mystic experience: the peak experience” and “philosophical, unhostile sense of humor,” which emphasize excellent personal features. The characteristics identified from this study emphasize individuals’ relationships with the country and family. Such as “loving their motherland and identifying with their culture,” “having a sense of responsibility and mission and being willing to dedicate oneself to the country at any time,” and “Showing filial respect to parents appropriately.” These characteristics are the direct expression of Chinese culture in terms of devoting oneself to the country and being filial to parents, which were not found in the results of Maslow’s study.

On the other hand, this study aligns more closely with research findings from African and Asian cultural backgrounds. For example, in the view of caregivers in Africa and Asia, mentally healthy individuals are people who contribute to the community and spend an enjoyable time in groups [28]. Thus, college students with good mental health can meet precise requirements at different levels: the individual and others, the individual and the family, and the individual and the nation, which is more of a relationship-oriented “big self” [62].

Thirdly, this research’s findings correspond with certain facets of the 24 character strengths and 6 virtues outlined in positive psychology, yet they also reveal disparities in specific aspects.

With the burgeoning of the positive psychology movement, some researchers have suggested that people with good mental health are not articulated merely as the absence of mental illness but as people who possess positive qualities, such as being highly resilient and well-being [63]. Seligman and colleagues summarized 6 virtues and 24 character strengths contributing to a good life [64], which have garnered wide attention. A point of convergence is that some positive psychological qualities emphasized by the characteristics identified in this study align with those highlighted in positive psychology. For instance, the characteristic of “being benevolent and kind” identified in this study emphasizes that mentally healthy college students are compassionate and kind. Similarly, one of the 6 virtues in positive psychology is humanity, which also focuses on kindness.

Nonetheless, notable distinctions exist between this study and the character strengths or virtues proposed by positive psychology. Firstly, in terms of the connotation of similar qualities, there are variations between the two. For example, the quality of “modesty” as a traditional Chinese virtue holds different implications than the Western perspective on “humility.” Modesty in Chinese culture carries much richer connotations than in the West, and core characteristics such as being open-minded, down-to-earth, and striving for improvement are unique to Chinese culture [65]. Additionally, while positive psychology views humility as an important but standalone character strength, this study found that mentally healthy college students are “being confident and also modest,” with modesty and confidence blending and coexisting harmoniously. This aligns with the encouragement of self-esteem, confidence, and self-improvement among the younger generation in China in recent years [66]. However, Chinese people still highly value modesty as a virtue while simultaneously emphasizing confidence. These seemingly contradictory qualities of confidence and modesty are valued, reflecting the dynamic balance of “yin and yang” in Chinese culture [67].

More importantly, this study has uncovered additional positive qualities beyond the 24 character strengths, such as “being inclusive and broad-minded”.These qualities carry strong moral attributes; in other words, possessing these moral qualities is essential for mental health. Confucianism emphasizes social morality, self-cultivation, and the development of a gentleman-like sage personality [68]. Self-cultivation is the basis for the ethical construction of family and society to perfect the ideal personality of governing the state and pacifying the world. The concept of “sageliness within and kingliness without” underscores this philosophy [69]. The characteristic “being inclusive and broad-minded” implies that mentally healthy college students exhibit tolerant and open-minded attitudes, embracing the principles of “Harmony, but Not Uniformity” and “The sea admits hundreds of rivers for its capacity to hold”(Chinese idioms) when encountering diverse viewpoints or adversity. Therefore, a mentally healthy college student possesses virtues such as tolerance and open-mindedness, showcasing solid moral values. In essence, college students’ mental health is intertwined with their moral attributes. A mentally healthy individual must embody essential moral qualities, which serve as markers of their overall well-being. Acknowledging the significance of moral virtues in defining and nurturing mental health among college students is crucial.

### Validation and extension to the theory of sociocultural models

Firstly, this study validates the theory of sociocultural models. On one hand, this study confirms how culture influences individual psychology as proposed in the theory of sociocultural models. In this study, psychological entities represent the characteristics of mentally healthy college students that guide their thoughts, behaviors, and attitudes. According to the findings of this study, Chinese traditional culture plays a significant role in shaping these characteristics. For example, the patriotic sentiments of important historical figures such as Wen Tianxiang and Yue Fei, as well as the thoughts of traditional Chinese culture such as “Death is not my concern should it benefit the country. How can I pick and choose for my loss or gains?” (Lin Zexu: *Two poems for family members on the way to the garrison*”) and “To ordain conscience for Heaven and Earth, to secure life and fortune for the populace, to carry on lost teachings of ancient sages, to build peace for posterity’ (Zhang Zai: *Heng Qu Yi Shuo*) are internalized in the characteristics of “Having a sense of responsibility and mission and being willing to dedicate oneself to the country at any time.” The country cultivates college students as pillars of talent, and Confucianism teaches “To be the first in the country to worry about the affairs of the state and the last to enjoy oneself.” (Fan Zhongyan: *The Yueyang Tower*). Thus, studying is not only for personal development but also for a sense of responsibility and contribution to the country, which arguably demonstrates the mental health characteristics of the specific group of college students with distinct traditional Chinese cultural connotations. Such findings align with the theory of sociocultural models, emphasizing how people internalize societal culture into their psychological entities to regulate their psychological activities.

On the other hand, this study validates how individual psychology externalizes and promotes the generation of new culture as proposed in the theory of sociocultural models. During China’s modernization, people have realized that only by daring to break through the shackles of existing ideas and exploring innovative development opportunities can the country move forward and develop sustainably. Many people have overcome difficulties and carried out the revolution, construction, and innovation in constructing Chinese socialism. Their love for the country and their sense of mission made them always meet the challenges of national reconstruction with high morale and perseverance [70]. Especially since the reform and opening-up, people’s minds have been fundamentally liberated, and the spring of scientific and technological progress has been ushered in. Their precious spiritual wealth, such as the characteristic of “daring to criticize, explore, and innovate,” has facilitated the development of new cultures like Chinese revolutionary and socialist cultures in modern times. Such findings align with the theory of sociocultural models, highlighting how group members externalize their psychological entities and transform them into new social cultures through social interactions and co-construction with existing social cultures.

Secondly, this study expands the content of the theory of sociocultural models. Due to a lack of specific pathways depicting the interaction between culture and psychology in the theory of sociocultural models, this study found that the significant carriers of interaction between culture and individual psychology are the spiritual world presented by historical and modern figures mentioned by the interviewees, as well as tangible worlds such as Chinese characters, poetry, martial arts, and art. These aspects of Chinese culture are internalized by college students as part of their psychological entities, guiding their words and actions and also shaping their perception of mental health. Conversely, the psychological entities of college students, such as the emergence of new concepts like “daring to criticize, explore, and innovate” in the construction of a new China, are transformed into emerging cultures, such as Chinese socialist culture through the role of figures like Qian Xuesen and stories as carriers.

### Strengths, limitations, and future research

This study possesses several strengths. Firstly, it is the first attempt to systematically explore the characteristics of college students’ mental health entirely based on Chinese culture. The 18 identified characteristics directly convey or reflect aspects of Chinese culture, significantly enriching the comprehension of college students’ mental well-being within the context of Chinese culture. Secondly, the study adheres to the elite principle research paradigm by using elite samples as participants. Consequently, the outcomes comprehensively delineate the characteristics of mentally healthy college students possessing an excellent psychological state rooted in Chinese culture. These findings not only provide an ideal model for nurturing mental health among college students but also engender fresh insights into mental well-being, culminating in a novel benchmark for mental health standards. Thirdly, this study delves into the unique characteristics of mentally healthy college students within Chinese culture from the students’ firsthand experiences. In contrast, prior scholars predominantly offered personal opinions on the characteristics of mentally healthy individuals based on their experiences, lacking the direct perspectives of college students.

This study also has some limitations. As a qualitative study, the nature of this research inherently limits the applications of its conclusions. Focused primarily on college students, generalizing the findings to other groups in China (such as civil servants) may be constrained. Moreover, this study exclusively examines Chinese college students without conducting cross-cultural research. The absence of direct comparative studies fails to highlight variations in mentally healthy characteristics across diverse cultures. For instance, the absence of a comparative study between Chinese and students from other cultures (such as American college students) hindered exploration into the distinctive characteristics and differences of mentally healthy college students from varying cultures. Consequently, extrapolating the results of this study to other cultural contexts also has its limitations. Despite some similarities between Chinese culture and certain cultures in Asia and Africa, direct inferences also have significant constraints.

Furthermore, in terms of understanding culture, there is no conclusive definition of what culture is and what Chinese culture is. Scholars have put forward many understandings and definitions of Chinese culture from different perspectives. Understanding and defining Chinese culture are still in the exploratory stage, which challenges this study. The researcher’s understanding and mastery of existing relevant knowledge are somewhat limited regarding the formation of research results and the depth of analysis and discussion.

Future research could consider the following aspects. Firstly, a comparative study of the characteristics of mentally healthy people in different cultural groups can be conducted. Since individualistic/collectivistic cultures influence Americans and Chinese to be more expressive of private selves/collective selves, and religious cultures also influence individual self-esteem [71] and form religious selves [72]. Therefore, some comparative studies with students from different cultural backgrounds can be conducted in the future. For example, a comparative study with three groups of college students from the United States, China, and India can be considered to compare whether there are differences in the characteristics of mentally healthy college students from different cultures. Secondly, some quantitative studies can be considered. For example, future research could refine specific characteristics identified in the study, like “being flexible and dialectical,” for more specific operational definitions and develop a scale to measure the mental health of different groups to validate how these characteristics are manifested in university students or other groups so that more further research could be conducted using this new scale, which may help facilitate replication of the findings. Thirdly, based on continuous learning and accumulation of Chinese culture, future research can do in-depth excavation and exploration of the manifestation and nature of these mental health characteristics. For example, future research could select the characteristics reflecting the culture of filial piety or Zhongyong culture and explore how these cultures change and develop into mental health characteristics with the development of science and technology, the change of social structure, and the collision of Chinese and Western cultures, which may also be of great significance.

### Practical implications

The Chinese culture has rich treasure resources and cultivated Chinese character traits, characteristics, and lifestyles. The results of this study show that many attitudes, ideas, and behaviors espoused by Chinese culture are manifestations of mental health. In particular, this study found the characteristics of mentally healthy college students based on Chinese culture, which is culturally applicable and more suitable for promoting the mental health of Chinese college students and can provide essential references and bases for mental health education and clinical practice.

On the one hand, this study can provide an overall theoretical framework for developing mental health courses for college students. Mental health courses are the most important and direct form of mental health education for college students in China, and they are also the primary way to improve the psychological quality of college students. The Ministry of Education requires colleges and universities to offer mandatory public courses on mental health for undergraduate students [73]. However, current mental health courses for Chinese college students rely mainly on Western mental health-related definitions, theories, and techniques for delivery [74, 75]. The five themes and 18 characteristics discovered in this study are systematic, providing a comprehensive and systematic theoretical basis for college students’ mental health courses.

In particular, the five themes discovered in this study—values pursuit, life attitude, interpersonal ideals, behavioral ability, and self-cultivation—can be employed as the central pillars for teaching and setting objectives in a college student mental health course rooted in Chinese culture. Furthermore, the 18 identified characteristics can form each lesson’s fundamental content and learning goals, establishing a comprehensive framework. For instance, the characteristics “being confident and also modest” can be one of the key topics under the theme of “self-cultivation.” By comparing Western views of mental health (focused on confidence) with Chinese beliefs (valuing both confidence and modesty) and blending students’ self-awareness with Chinese cultural insights, the course can delve into the importance of confidence and modesty in Chinese culture. Strategies for cultivating these characteristics can be discussed, shedding light on the unique aspects of mental health development among college students within Chinese cultural contexts.

Secondly, this research offers valuable insights for fostering healthy personalities among college students in psychological counseling methods from the perspective of Chinese culture. On the one hand, this study has a guiding significance for setting goals in psychological counseling. Psychological counseling has traditionally emphasized decreasing negative emotions and boosting positive ones. Nevertheless, this study serves as a reminder for counselors to reassess this counseling objective. Throughout the counseling process, counselors should not only focus on diminishing negative emotions but also be wary of potential complications stemming from excessive positive emotions, stressing the importance of a moderate expression of positive and negative emotions.

On the other hand, the discoveries of this study could serve as a wellspring of inspiration for crafting indigenous approaches to psychological counseling. This research reveals that mentally healthy college students possess the characteristic “possessing good qualities advocated by Confucianism, Buddhism, and Taoism coexist harmoniously.” Within Chinese culture, the symbiotic interplay among Confucianism, Buddhism, and Taoism stands out as a cornerstone [76], where these philosophies coexist compatibly and mutually influence each other in shaping Chinese characters [77]. Future scholars might devise counseling methodologies rooted in the principle of harmonious coexistence found within Confucianism, Buddhism, and Taoism, potentially empowering individuals to bolster their mental health through these culturally embedded psychological counseling approaches.

## Conclusion

This study explores the characteristics of mental health of college students with good psychological states from the perspective of Chinese culture and finds 18 characteristics, based on which five themes are formed: value pursuit, life attitude, interpersonal ideal, behavior ability, and self-cultivation. The 18 characteristics are typical of Chinese culture or its features, focusing on multi-level relationships with others, parents, and the country. They are also typical of Chinese culture with moral attributes, an emphasis on self-cultivation, a balance of Yin and Yang, and the coexistence of Confucianism, Buddhism, and Taoism. These findings help enrich the research on culture and mental health, highlight the Chinese cultural connotations of mental health, and help form an ideal standard of mental health for college students. Findings can serve as a theoretical foundation for improving the mental well-being of Chinese college students, act as a guiding light for enhancing students’ mental health, and be integrated directly into the mental health curriculum as course content. Mental health education activities based on these findings can help promote, maintain, and cultivate college students’ mental health literacy and healthy personalities to fulfill their potential and become the pillars of the nation.

### Supplementary Information

**Supplemenatary Material 1. **

## Data Availability

The datasets for this study are not readily available because they consist of interview data, for which confidentiality cannot be safeguarded. Therefore, the data will not be made available. Requests to access the datasets should be directed to XJ, jiaxiaoming@bit.edu.cn.
