# Supplementary material for: How would you describe a mentally healthy college student based on Chinese culture? A qualitative research from the perspective of college students
Source: BMC Psychol. 2024 Apr 15;12:207. doi: 10.1186/s40359-024-01689-7 (PMC11020864; doi:10.1186/s40359-024-01689-7)
Supplement: Supplementary file 1 — Supplemenatary Material 1. [file 40359_2024_1689_MOESM1_ESM.docx]

***Appendix 1：****Semi-structured interview guide（Pre-interview）.*

| 1 | What do you think is mental health? What do you think a “mentally healthy” college student is like? You can use yourself or your classmates as examples. |
| --- | --- |
| 2 | What do you think is Chinese culture? What is your understanding of Chinese culture? |
| 3 | What do you think is related to college students’ mental health in Chinese culture? |
| 4 | What makes you rate yourself a score of “*” on mental health? In comparison with yourself, do you have anything to add to “what a ‘mentally healthy’ college student is like”? |
| 5 | What specific elements of Chinese culture reflect your mental health? How do you understand it? |

***Appendix 2：****Formal* *semi-structured interview guide.*

| 1 | What do you think is mental health? What do you think a “mentally healthy” college student is like? You can use yourself or your classmates as examples. |
| --- | --- |
| 2 | What do you think is Chinese culture? What is your understanding of Chinese culture? |
| 3 | What a “mentally healthy” college student is like based on Chinese culture? You can take yourself or your classmates as an example. |
| 4 | What makes you rate yourself a score of “*” on mental health? In comparison with yourself, do you have anything to add to “what a ‘mentally healthy’ college student is like”? |
| 5 | What specific elements of Chinese culture reflect your mental health? How do you understand it? |
